# Common brain areas engaged in false belief reasoning and visual perspective taking: a meta-analysis of functional brain imaging studies

**DOI:** 10.3389/fnhum.2013.00712

**Published:** 2013-11-01

**Authors:** Matthias Schurz, Markus Aichhorn, Anna Martin, Josef Perner

**Affiliations:** ^1^Center for Neurocognitive Research, University of SalzburgSalzburg, Austria; ^2^Department of Psychology, University of SalzburgSalzburg, Austria

**Keywords:** neuroimaging meta-analysis, theory of mind, false belief, visual perspective taking, temporo-parietal junction

## Abstract

We performed a quantitative meta-analysis of functional neuroimaging studies to identify brain areas which are commonly engaged in social and visuo-spatial perspective taking. Specifically, we compared brain activation for visual-perspective taking to activation for false belief reasoning, which requires awareness of perspective to understand someone's mistaken belief about the world which contrasts with reality. In support of a previous account by Perner and Leekam (2008), our meta-analytic conjunction analysis found common activation for false belief reasoning and visual perspective taking in the left but not the right dorsal temporo-parietal junction (TPJ). This fits with the idea that the left dorsal TPJ is responsible for representing different perspectives in a domain-general fashion. Moreover, our conjunction analysis found activation in the precuneus and the left middle occipital gyrus close to the putative Extrastriate Body Area (EBA). The precuneus is linked to mental-imagery which may aid in the construction of a different perspective. The EBA may be engaged due to imagined body-transformations when another's viewpoint is adopted.

## Introduction

Being able to adopt another person's perspective is an important feature of human social cognition. In the last decade and a half, functional neuroimaging studies have sought to identify the neural mechanisms underlying this ability. Two lines of research have emerged. One group of studies has looked at perspective relevant processes in the context of visuo-spatial cognition, typically by asking about the visual experience arising from a different point of view (visual perspective taking). Studies in this field can be divided into level 1 and 2 visual perspective taking (Masangkay et al., 1974; Flavell et al., 1981). Level 1 perspective taking refers to the ability to distinguish what people can and cannot see, e.g., that two persons looking at different sides of a piece of paper see different things. Level 2 perspective taking refers to the ability to understand that when two persons look at an object from different viewpoints or angles, they arrive at different and maybe contradictory descriptions. Besides research on visual perspective taking, another group of studies has looked at perspective relevant processing in social contexts. The terms “mentalizing”, “mind reading” or “theory of mind” refer to our ability to think about the mental states—such as thoughts and beliefs—of ourselves and others (Premack and Woodruff, 1978). A way to test children's ability to attribute mental states to others is the false belief task. Children are told a story in which a character, Mistaken Max, fails to witness how his chocolate is unexpectedly transferred from one location to another. Therefore, he believes that the chocolate is still in its original location. Children have to predict whether he will look for the chocolate in its original or in its new location. To arrive at the correct answer—its original location—children have to take into account that Mistaken Max holds a false belief about the location of the chocolate, which contrasts with their own knowledge about its real location.

Developmental research showed that the ability to make correct level 2 visual perspective judgments emerges about 2 years later than the ability to master these judgments at level 1 (Masangkay et al., 1974). At the same time when children start to master level 2 judgments—at around 4 to 5 years of age—they also start to pass the false belief test (Wimmer and Perner, 1983). Hamilton et al. (2009) found that theory of mind performance (assessed with a set of tasks including the false belief task) significantly predicted performance on a level 2 visual perspective task in a sample of 4–8 year old children. In contrast, neither performance on a mental rotation task nor children's verbal mental age showed a relation to level 2 visual perspective taking. One explanation (e.g., Perner et al., 2003; Perner and Rössler, 2012) for this link between level 2 visual perspective and false belief understanding is that both tasks require an understanding of perspective, i.e., that different persons can have different views on/or beliefs about one and the same state of affairs. In addition, for both tasks children must be able to intentionally switch to another perspective.

Brain activation for false belief reasoning was mostly studied by presenting short stories to adult participants, and results show a consistent network of brain areas activated (see e.g., Saxe and Kanwisher, 2003; Perner et al., 2006; Saxe and Powell, 2006), including the medial prefrontal cortex (mPFC), bilateral temporal poles, the precuneus, and bilateral temporo-parietal junction (TPJ) areas. The mPFC was linked to the processing of socially and emotionally relevant information about other people that is contained in the stories, but not specifically linked to the processing of belief (Aichhorn et al., 2006; Saxe, 2006; Saxe and Powell, 2006). For example, an fMRI study found that the mPFC was equally engaged by stories about a person's thoughts and by stories about a person's physical appearance or bodily sensations (Saxe and Powell, 2006). The temporal poles were linked to the retrieval of social semantic knowledge from long-term memory, which takes place because participants read stories about persons in social situations (e.g., Gallagher and Frith, 2003; Ross and Olson, 2010). Based on its engagement in visuo-spatial mental imagery (e.g., Ghaem et al., 1997; Hanakawa et al., 2003), it was assumed that the precuneus subserves mental imagery to represent another person's perspective in theory of mind tasks (Cavanna and Trimble, 2006). Similarly, the TPJ areas were linked to the representation of mental and non-mental perspectives (Perner et al., 2006; Perner and Leekam, 2008). The right TPJ was specifically linked to the representation of beliefs, as it was found to respond more strongly when reading statements about a person's thoughts than when reading statements about physical appearance or bodily sensations (Saxe and Powell, 2006), and also compared to reading statements about a person's emotions and perceptions (Zaitchik et al., 2010). In another study, an interesting observation was made for the left TPJ. Perner et al. (2006) presented a novel condition—false sign stories—in addition to the standard false belief and photo control stories. An example for a false sign story is: “The sign to the monastery points to the path through the woods. While playing, the children make the sign point to the golf course. According to the sign the monastery is now in the direction of the … golf course / woods”. False sign stories present a very different problem than false belief stories which require figuring out an internal and unobservable mental state of another person. False sign stories simply require reflecting upon the external and directly observable world—the direction to where a sign is pointing. Nevertheless, Perner et al. (2006) found equally high activation for false sign stories as for false belief stories in the left TPJ (but not in the right TPJ). A significantly lower level of activation was found for photo control stories. These findings suggest that the left TPJ is responsible for an operation that is common to reasoning about false belief and false signs: processing of a perspective difference, regardless of whether it is an unobservable inner state (belief) or a visible state (where the sign points). Both in the case of false belief stories and false sign stories, two contrasting perspectives of one and the same state of affairs are involved: the belief of a person that contrasts with one's own knowledge of reality, or the location to which a sign is pointing that contrasts with one's own knowledge about the real location of a target.

In comparison to the detailed picture that has already emerged for the neural correlates of false belief reasoning, brain imaging evidence on visual perspective taking is relatively scattered and has been discussed less extensively. To our knowledge, no systematic review or meta-analysis of visual perspective studies has been done yet. When contrasting judgments about another person's perspective with judgments about one's own perspective (level 1 and 2 taken together) studies mainly found activation in three areas: (i) Lateral prefrontal cortices (e.g., Vogeley et al., 2004; Aichhorn et al., 2006; David et al., 2006, 2008; Dumontheil et al., 2010; Mazzarella et al., 2013), (ii) bilateral parietal and temporo-parietal areas (Vogeley et al., 2004; David et al., 2006, 2008; Kaiser et al., 2008; Mazzarella et al., 2013) and (iii) the precuneus (Vogeley et al., 2004; Kaiser et al., 2008; Dumontheil et al., 2010). The lateral prefrontal cortices – in particular the inferior frontal gyri—are engaged by cognitive control in interference tasks, such as the color-word Stroop task or stimulus-response reversal studies (for review see Derrfuss et al., 2005). Likewise, researchers linked these areas to the inhibition of the irrelevant own perspective when making visual perspective judgments (McCleery et al., 2011; Ramsey et al., 2013). Activation in temporo-parietal areas was linked to the representation of perspectives, and in particular to the representation of differences in perspective and ownership of perspective (McCleery et al., 2011). In addition, Ramsey et al. (2013) suggested that superior parietal areas are engaged in perspective selection, i.e., in choosing the relevant over the irrelevant perspective. This was assumed to take place in cooperation with lateral prefrontal areas, forming a functional network that is sometimes referred to as the “fronto-parietal control network” (e.g., Vincent et al., 2008). The precuneus was rarely mentioned when discussing the neurocognitive processes subserving visual perspective taking, although it is implicated in multiple forms of visuo-spatial mental imagery (e.g., Ghaem et al., 1997; Hanakawa et al., 2003).

Our review of the neuroimaging literature on false belief reasoning and visual perspective taking showed that both discussed the left TPJ and the precuneus as candidate areas for representing perspectives and perspective differences. Only little functional imaging research has addressed this connection. To our knowledge, no study has directly compared activation for false belief reasoning to activation for visual perspective taking. Aichhorn et al. (2006) measured brain activation for level 2 visual perspective taking, and asked participants to judge the spatial arrangement of two objects (e.g., “the block is in front of the pole”) from the viewpoint of an avatar. The authors found brain activation for level 2 perspective taking—compared to making the same judgments from one's own viewpoint—in an area of the left TPJ that was also activated in a number of earlier studies on theory of mind (e.g., Gallagher et al., 2000; Ruby and Decety, 2003; Saxe and Kanwisher, 2003). Aichhorn et al. (2006) therefore concluded that the left TPJ is responsible for representing different perspectives and is commonly engaged by tasks which require such processing. However, a more recent study provided evidence against this interpretation. David et al. (2008) asked participants to either make a visual perspective or a mentalizing (preference) judgment with respect to two objects in front of an avatar. The avatar was facing participants. In the level 2 perspective judgment, participants were asked which of the two objects (left or right) was elevated from the avatar's point of view. For example, if the elevated object was on the left from the avatar's point of view, this implied that it was shown on the right side of the image to participants. In the preference judgment, participants were asked to judge which object the avatar would prefer—based on his gestures (e.g., pointing at one object) and facial expression. During the judgments participants always indicated the object as seen from their own perspective. The comparison of brain activation between the tasks showed two completely distinct networks of brain activation and no overlap in the left TPJ. Therefore, David et al. (2008) concluded that visual perspective taking and mentalizing rely on different cortical mechanisms.

Studies that compared brain activation for visual perspective taking and mentalizing show contradictory results. However, these studies never directly compared activation for false belief reasoning with visual perspective taking. As we have outlined above, both developmental research and neurocognitive theories speak for a functional link between these tasks. The present study evaluates the functional overlap between false belief reasoning and visual perspective taking by means of a quantitative meta-analysis of brain imaging studies. To increase statistical power, we analyze both level 1 and 2 visual perspective taking studies in our meta-analysis. Based on the reviewed literature, we expect to find a functional overlap in the left TPJ and in the precuneus.

## Methods

We performed key-word searches in the databases PubMed, Science Citation Index, and PsycInfo. The first criterion of our search was that studies included one of the key-words “neuroimaging” or “fMRI” or “PET”. For our false belief meta-analysis, the second criterion was that studies further included the key-words “false belief” or “theory of mind”. For our visual perspective taking meta-analysis, the second criterion was that studies included the key-words “perspective taking” or “visual perspective” or “viewer rotation”^1^. In a second step, we extended our literature samples by searching the reference lists of recent meta-analyses on theory of mind and social cognition (Mar, 2011; Bzdok et al., 2012; Denny et al., 2012; Murray et al., 2012) as well as the reference lists of most recent publications on visual perspective taking (Lambrey et al., 2012; Mazzarella et al., 2013; Ramsey et al., 2013).

**Figure 1:**
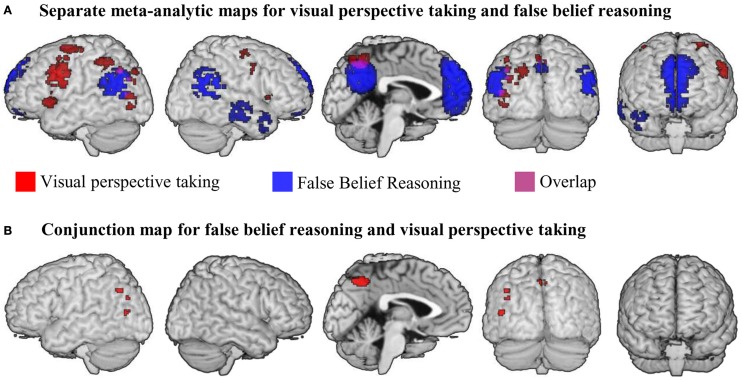
**(A)** Results of meta-analyses for false belief reasoning (blue) and visual perspective taking (red). Overlap between result maps is shown in purple. **(B)** Results of a conjunction analysis searching for brain areas active for false belief reasoning AND visual perspective taking. All maps were thresholded at voxel-wise threshold of *p* < 0.005 uncorrected and a cluster extent threshold 10 voxels.

We then applied a number of methodological selection-criteria to the literature identified by our search (see e.g., Radua et al., 2012). Studies were only selected if they had performed a whole brain analysis and reported activation coordinates in standard space (MNI or Talairach). We ensured that the same threshold throughout the whole brain was used within each included study, in order to avoid biases toward liberally thresholded brain regions. This does not mean that different studies should employ the same threshold. We included 25 studies (*N* = 419) in our meta-analysis on false belief and 14 studies (*N* = 216) in our meta-analysis on visual perspective taking. We used Effect-Size Signed Differential Mapping (ES-SDM) software, version 2.31 for meta-analysis (Radua et al., 2010, 2012; http://www.sdmproject.com). ES-SDM uses standard effect size and variance-based meta-analytic calculations. Based on the reported *t*-values and the sample size of a study, ES-SDM creates a map of effect-sizes (Hedge's *g* values) and their variances. Variance is estimated from the map of effect-sizes and the sample size of the study. Effect- sizes are exactly calculated for those voxels containing a peak reported in the results table of an original study. For the rest of the voxels, an effect-size is estimated depending on the distance to close peaks (<20 mm) by means of an unnormalized Gaussian kernel. In the present analysis, we used the recommended Gaussian kernel with a FWHM of 20 mm. A validation study which compared the results of coordinate based ES-SDM meta-analysis to the results of a standard voxel-wise GLM analysis of the same original data (Radua et al., 2012) found that this FWHM provided an optimal balance between sensitivity and specificity. For statistical-analysis, all foci were transformed to Talairach space which is the native space of the software, by using the matrix transformations proposed by Lancaster et al. (2007). We calculated a mean analysis for each task-group. Calculation of the meta-analytic mean map is implemented by a random-effects model in which each study is weighted by the inverse of the sum of its variance plus an estimate of between-study heterogeneity. The latter is obtained by the DerSimonian-Laird method (DerSimonian and Laird, 1986). This approach enables studies with larger sample size or lower variability to contribute more and that effects are assumed to randomly vary between samples. The statistical significance was assessed by a permutation test; 100 random maps were generated with the same number of input foci as included in the to-be-tested map (see Radua et al., 2012). Finally, the meta-analytic maps were thresholded using a voxel-level (height) threshold of *p* < 0.005 (uncorrected) and a cluster-level (extent) threshold of 10 voxels. This uncorrected threshold was found to optimally balance sensitivity and specificity, and to be an approximate equivalent to a corrected threshold of *p* < 0.05 in original neuroimaging studies (Radua et al., 2012). We performed a conjunction analysis (see Figure 1B) with the “image calculator” utility in SPM8 (www.fil.ion.ucl.ac.uk). Conjoint activation is determined by a voxel-wise combination of results by a logical AND function. For convenience, we report all activations in MNI-space.

## Results

### False belief reasoning

Studies on false-belief reasoning mainly used two types of tasks. One group of studies contrasted stories about false belief with stories about an outdated photograph. We give some examples in Table 1. In total, we found 15 studies (reported in 14 publications) that relied on this type of contrast (Saxe and Kanwisher, 2003; Saxe and Wexler, 2005; Perner et al., 2006; Saxe and Powell, 2006; Saxe et al., 2006; Young et al., 2007, 2010, 2011; Kliemann et al., 2008; Mitchell, 2008; Aichhorn et al., 2009; Young and Saxe, 2009; Dodell-Feder et al., 2011; Lee et al., 2011). In the false belief story a short text passage is presented, which involves a person holding a false belief. A test question asks participants about the belief or its behavioral consequences. In the control task, a short text passage describes a photograph (or a similar physical representation) of the past, together with a note about how things shown on the photograph have changed by now. Participants are asked what is shown on the photo. Another more heterogeneous group of studies presented similar stories about false belief. In this group of studies, however, stories of different length and richness were presented, and different types of control stories were used. We give some examples for false belief studies of our second group in the lower part of Table 1. The common element of these studies is that they present a story (sentence or cartoon format) about a person that holds a false belief as activation tasks. Participants are asked a question which relates to the false belief of the person. In the control condition, again a story about a person is presented, but here the person does not hold a false belief. Participants are asked about non-mental state information in the story. In total, we found 10 studies that relied on this type of contrast (Fletcher et al., 1995; Happé et al., 1996; Gallagher et al., 2000; Nieminen-von Wendt et al., 2003; Hynes et al., 2006; Kobayashi et al., 2006, 2007; Gobbini et al., 2007; Abraham et al., 2010; Jimura et al., 2010). We pooled the two groups of tasks that present stories about false belief into one single meta-analysis (total *n* = 25).

**Table 1:** **Examples for false belief reasoning tasks**.

**Author**	**Activation task**	**Control task**
**FALSE BELIEF VIGNETTES VS. PHOTO CONTROL VIGNETTES (3 EXAMPLES OUT OF 15 STUDIES)**		
Aichhorn et al., 2009	Read a short vignette involving a person holding a false belief. Predict the behavior of that person based on her belief. e.g.: *‘Julia sees the ice cream van go to the lake. She doesn't see that the van turns off to the town hall. Therefore, Julia will look for the ice cream van at the … ?’ (lake or town hall)*.	Read a short vignette involving a photograph of the past, and a description how things shown on the photo have changed by now. Answer a question about the outdated scene shown on the photo. e.g.: *‘Julia takes a picture of the ice-van in front of the pond. The ice cream van changes to the market place; the picture gets developed. On the picture, the ice-van is at the … ?’ (pond or market place)*.
Saxe and Kanwisher, 2003	Read a short vignette involving a person holding a false belief. Answer a question about her belief. e.g.: *‘John told Emily that he had a Porsche. Actually, his car is a Ford. Emily doesn't know anything about cars so she believed John. When Emily sees John's car, she thinks it is a … ?’ (Porsche or Ford)*.	Read a false-photograph vignette. Answer a question concerning the outdated content in the photo. e.g.: *‘A photograph was taken of an apple hanging on a tree branch. The film took half an hour to develop. In the meantime, a strong wind blew the apple to the ground. The developed photograph shows the apple on the … ?’ (tree or ground)*.
Lee et al., 2011	Read a short vignette involving a person holding a false belief. Answer a question about her belief. e.g.: *‘David knows that Ethan is very scared of spiders. Ethan, alone in the attic, sees a shadow move and thinks it is a burglar. David hears Ethan cry for help. David assumes that Ethan thinks he has seen … ?’ (a spider or a burglar)*.	Read a false-photograph vignette. Answer a question concerning the outdated content in the photo. e.g.: *‘Amy made a drawing of a treehouse three years ago. That was before the storm. We built a new treehouse last summer, but we painted it red instead of blue. The treehouse in Amy*'*s drawing is … ?’ (red or blue)*.
**FALSE BELIEF STORIES VS. CONTROL STORIES (2 EXAMPLES OUT OF 10 STUDIES)**		
Abraham et al., 2010	Read a story describing a character's mental state (belief/desire) about an object. Then the object in reality is described. Indicate if the character is surprised/delighted by the reality. e.g.: *‘Thomas believes that there will be a lot of sugar in the chocolate pudding.–The chocolate pudding does not taste sweet.–Would that surprise Thomas?’ (yes or no)*.	Read a story describing a non-mental attribute of a group of persons. Then a particular member of this group is introduced. Indicate if that member holds the non-mental attribute (syllogistic reasoning).e.g.: *‘All students at the dance academy own more than three pairs of dance shoes.–Sonja studies dance at the academy.–Does Sonja own more than three pairs of dance shoes?’(yes or no)*.
Fletcher et al., 1995	Read a story about a character performing an action. The reason for that action must be inferred from his/her false belief (sometimes also ignorance). Explain (silently) why he did that. e.g.: *‘A burglar who has just robbed a shop is making his getaway. As he is running home, a policeman on his beat sees him drop his glove. He doesn't know the man is a burglar, he just wants to tell him he dropped his glove. But when the policeman shouts out to the burglar, “Hey, you! Stop!”, the burglar turns around, sees the policeman and gives himself up. He puts his hands up and admits that he did the break-in at the local shop.’*	Read a story about a character performing an action. The reason for that action can be inferred from the information in the story and is not related to false belief. Explain (silently) why he did that. e.g.: *‘A burglar is about to break into a jewelers’ shop. He skillfully picks the lock on the shop door. Carefully he crawls under the electronic detector beam. If he breaks this beam it will set off the alarm. Quietly he opens the door of the store-room and sees the gems glittering. As he reaches out, however, he steps on something soft. He hears a screech and something small and furry runs out past him, toward the shop door. Immediately, the alarm sounds.’*

We performed a meta-analysis on the reported activation maps for the contrast false belief stories > control stories. Results are shown in blue in Figure 1A and are listed in Table 2. The largest cluster of meta-analytic convergence was found in the mPFC, including parts of dorsal and ventral mPFC and the anterior cingulate gyrus. Another large cluster of convergence was found in precuneus and posterior cingulate gyrus bilaterally. Further clusters of convergent activation were found in bilateral temporo-parietal areas, spanning across parts of middle and superior temporal gyri up to the inferior parietal lobule (up to *z* = 42). Two smaller clusters of convergence were found in anterior parts of the right temporal lobe.

**Table 2:** **Results of meta-analyses for False Belief Reasoning and Visual Perspective Taking**.

			**Cluster center**						**Individual foci**			
#	**Hem**	**Label**	***x***	***y***	***z***	**BA**	***z*-val**.	**vx**	***x***	***y***	***z***	**Label**
**FALSE BELIEF REASONING (25 STUDIES)**												
1	R	Precuneus	6	−59	35	7	5.00	1282	4	−42	33	R cingulate
									0	−72	38	L cuneus
									0	−59	35	L precuneus
									0	−42	35	L cingulate
2	R	Supramarginal	62	−45	21	40	7.70	886	55	−67	15	R mid. temporal
									47	−61	39	R angular
									56	−56	25	R sup. temporal
									58	−52	42	R inf. parietal
3	R	Sup. temporal	51	−9	−9	22	3.21	326	61	−23	−17	R mid. temporal
									61	−16	−15	R mid. temporal
4	R	Sup. temporal	46	11	−24	38	2.606	207	48	8	−33	R sup. temporal
									40	11	−22	R inf. frontal
5	L	Mid. temporal	−57	−65	27	39	6.58	917	−50	−61	45	L inf. parietal
									−44	−61	40	L angular
									−59	−56	29	L sup. temporal
									−55	−47	28	L supramarginal
6	L	Sup. frontal	−5	60	21	9	6.42	2083	−18	47	29	L sup. frontal
									−5	60	28	L med. frontal
									3	48	−7	R ant. cingulate
									4	50	33	R med. frontal
									6	58	23	R sup. frontal
**VISUAL PERSPECTIVE TAKING (14 STUDIES)**												
1	R	Precentral	41	−8	54	4	2.69	72	58	1	32	R precentral
2	R	Insula	44	15	2	13	2.53	21				
3	L	Inf. occipital	−48	−80	2	18	2.74	71	−46	−77	11	L mid. occipital
4	L	Mid. temporal	−39	−73	32	39	2.53	101	−31	−81	33	L sup. occipital
									−18	−63	40	L precuneus
5		Precuneus	0	−53	52	7	2.76	187	0	−66	54	precuneus
									4	−54	50	R precuneus
6	L	Cerebellum	−27	−53	−35		2.73	175	−23	−55	−28	L cerebellum
									−24	−59	−38	L cerebellum
7	L	Inf. parietal	−37	−43	47	40	2.95	252	−41	−59	42	L angular
									−37	−60	33	L mid. temporal
8	L	Mid. frontal	−42	15	33	9	3.60	1054	−33	−5	62	L precentral
									−42	5	34	L precentral
									−38	7	13	L insula
									−42	8	27	L inf. frontal
									−44	14	−5	L insula
**FALSE BELIEF and VISUAL PERSPECTIVE TAKING**												
1	R	Precuneus	4	−54	50	7	2.66	46	6	−56	46	R precuneus
2	L	Mid. temporal	−39	−73	32	39	2.49	4				
3	L	Mid. occipital	−49	−72	13	37	2.32	12				
4	L	Angular	−41	−59	42	39	2.46	19	−41	−63	40	L angular
5		precuneus	0	−53	52	7	2.76	63	0	−66	54	precuneus
									−5	−51	52	L precuneus

### Visual perspective taking

Compared to the large number of imaging studies on false belief reasoning, relatively few imaging studies on visual perspective taking exist. We identified three groups of visual perspective tasks in the literature: level 1 visual perspective taking (3 studies), level 2 visual perspective taking (5 studies), and level 2 imagined viewer rotation (6 studies). Due to the small sample-sizes of these task-groups, it was not possible to perform individual meta-analyses. We therefore decided to merge the different visual perspective tasks into a pooled analysis, which gave us a large enough sample for quantitative meta-analytic calculations (*n* = 14). Later on (see section Region of interest based review), we provide a complementary results overview for individual task-types.

Table 3 gives task-descriptions for all visual-perspective taking studies in our meta-analysis. Level 1 visual perspective taking studies typically present a scene with an avatar and a number of objects. Participants are asked how many of these objects the avatar can see (while some of the objects are behind the avatars' back). In the control conditions of level 1 visual perspective taking studies, participants are asked how many objects they can see themselves^2^. Level 2 visual perspective taking tasks also typically present a scene with an avatar and a number of objects. However, here the avatar is able to see all of the objects in the scene, but views them from a different angle. Participants are asked to indicate the relative position of one object from the avatar's viewpoint. In the control condition of level 2 visual perspective tasks, participants are asked about the relative location of one object from their own perspective. The last type of visual-perspective taking tasks in our meta-analysis, level 2 imagined viewer rotation tasks, typically present an array of objects and ask to imagine viewing this array from a different angle. Then, participants are asked to indicate the relative position of one object from the imagined viewpoint. Two types of control tasks are frequently used in studies on imagined viewer rotation. In one type of control task, participants have to indicate the relative position of one object in the array as seen from their actual viewpoint (similar to the control conditions in level 2 visual perspective taking tasks). In another type of control task, a so-called object rotation task, participants are asked to imagine rotating the array around its vertical axis (e.g., with their right hand), and then indicate the current position of one object from their viewpoint.

**Table 3:** **ROI-based follow-up review: + signs denote that a study reported activation within 20 mm distance to a peak of our meta-analytic conjunction (20 mm corresponds to the smoothness of meta-analysis)**.

**First author**	**PREC**	**ANG**	**OCC**	**Persp. task**	**Control task**
**LEVEL 1 VISUAL PERSPECTIVE TAKING**					
Vogeley et al., 2004	+	+		You see a scene including an avatar and a number of objects. Indicate how many objects the avatar can see.	You see a scene including an avatar and a number of objects. Indicate how many objects you see.
Kaiser et al., 2008	+	+		You see a scene including an avatar and a number of objects. Indicate how many objects the avatar can see.	You see a scene including an avatar and a number of objects. Indicate how many objects you see.
Dumontheil et al., 2010^*^	+		+	You see a scene including an avatar and a number of objects. Follow the avatar's instruction (e.g., ‘move the large object up’). This instruction is dependent on the avatar's perspective (e.g., he can't see the largest object in the scene).	You see a scene including an avatar and a number of objects. Follow the avatar's instruction (e.g., ‘moves the large object up’). As a rule, if the avatar has a male (female) voice, you can only move certain objects.
**LEVEL 2 VISUAL PERSPECTIVE TAKING**					
Aichhorn et al., 2006				You see a scene including an avatar and two objects. Indicate their relative spatial arrangement e.g., 'Block is in front of the pole' from the viewpoint of an avatar.	You see a scene including an avatar and two objects. Indicate their relative spatial arrangement e.g., ‘Block is in front of the pole’ from your own viewpoint.
David et al., 2006		+		You see a scene including two avatars facing you. You and the avatars play a ball-tossing game. Take the perspective of one of them and indicate in which direction (left or right) he must throw the ball to pass it to the other avatar.	You see a scene including two avatars facing you. You and the avatars play a ball-tossing game. Indicate from your own perspective in which direction (left or right) you must throw the ball to pass it to one of the avatars.
David et al., 2008		+		You see a scene including an avatar facing you and two objects, located between you and the avatar. Indicate from his perspective which object (left or right) is elevated.	You see a scene including an avatar facing you and two objects, located between you and the avatar. Indicate from your perspective which object (left or right) is elevated.
Kockler et al., 2010			+	You see a scene including an avatar and one object. Indicate from the avatar's perspective if the object is to his left or right.	You see a scene including an avatar and one object. Indicate from your own perspective if the object is to your left or right.
Mazzarella et al., 2013		+		You see a table with an object on it. An avatar stands next to the table. Indicate from his perspective if the object is to his left or right.	You see a table with an object on it. An avatar stands next to the table. Indicate from your perspective if the object is to your left or right.
**LEVEL 2 IMAGINED VIEWER ROTATION**					
Creem et al., 2001	+			You see an array of four objects. Imagine being located in the array's center. Then imagine that your body position is rotated to a certain degree. Perform a relative location judgment (which object is on your right?).	You see an array of four objects. Imagine being located in the array's center. Stick to your actual body orientation, do not imagine a rotation. Perform a relative location judgment (which object is on your right?).
Zacks et al., 2003		+	+	You see an array of four objects. Imagine viewing the array from a different angle (i.e., imagine a self-rotation around the array). Indicate if a particular object is now on the left or right side of the array.	You see an array of four objects. Imagine that the array rotates along its vertical axis, while your own position remains the same. Indicate if a particular object is now on the left or right side of the array.
Wraga et al., 2005			+	You see a Shepard-Metzler object. Imagine viewing the object from a different angle (i.e., imagine a self-rotation around the array). Indicate if you now can see a particular side of the object.	You see a Shepard-Metzler object. Imagine rotating the object along. Indicate if you now can see a particular side of the object.
Creem-Regehr et al., 2007	+		+	You see an array of 6 objects surrounding a hand. Imagine viewing the hand from the position of one of the objects (i.e., imagine a self-rotation to this position). Indicate if the thumb is now to your left or right.	You see an array of 6 objects surrounding a hand. Indicate from your actual viewpoint if the thumb is to your left or right.
Wraga et al., 2010	+	+	+	You see a Shepard-Metzler object. Imagine viewing the object from a different angle (i.e., imagine a self-rotation around the array). Indicate if you now can see a particular side of the object.	You see a Shepard-Metzler object. Imagine holding the object in the right hand and rotating it in a specific way. Indicate if you now can see a particular side of the object.
Lambrey et al., 2012				You see a table with objects on it. Imagine rotating yourself around it to the position of an avatar / arrow (activation collapsed). Memorize the arrangement of objects from this perspective (tested later).	You see a table with objects on it. Imagine rotating the table until one object is in front of an avatar / arrow (activation collapsed). Memorize the current arrangement of objects from your own perspective (tested later).

PREC … Precuneus x = 0, y = −53, z = 52; ANG … Angular Gyrus x = −41, y = −59, z = 42; OCC … Middle Occipital Gyrus x = −49, y = −72, z = 13;

* Dumontheil et al.’s (2010) study could also be classified as a level 2 perspective task. The picture stimuli used in the task show a level 1 perspective difference. However, the task also presents statements (e.g., “move the large ball up”) that have to be interpreted from another person's perspective. A correct interpretation requires understanding that the other person has a different perspective of the entire scene (from his perspective, one particular ball is the largest of all, whereas from one's own point of view, another ball is the largest of all).

We performed a meta-analysis on the reported activations for all three types of visual perspective taking compared to their respective control condition. Figure 1A shows clusters of reliable meta-analytic convergence for visual perspective taking in red, and results are listed in Table 2. The largest cluster of convergent activation was found in the left lateral prefrontal cortex, with its peak in the left middle frontal gyrus. The cluster further included parts of the inferior frontal gyrus, the insula and the precentral gyrus. In the right hemisphere, lateral prefrontal activation was substantially smaller compared to the left. Two small clusters of activation were found, located in the right precentral gyrus and right insula. Larger clusters were found in the left inferior parietal lobule and in the precuneus. The left inferior parietal cluster included parts of the angular gyrus and the posterior middle temporal gyrus. The precuneus cluster spanned both hemispheres. In addition to the left inferior parietal area, two other clusters of convergence were found in left temporo-parietal areas. One was located in the left posterior middle temporal gyrus extending into the superior occipital gyrus; the other located in the left inferior and middle occipital gyri, near the location of the Extrastriate Body Area (EBA, Downing et al., 2001). Finally, a cluster of convergent activation was found in the left cerebellum (not visible in Figure 1A because of its location buried underneath the cerebellar surface).

### Conjunction analysis

Our conjunction analysis determined which brain areas showed convergent activation for both false belief reasoning and visual perspective taking. Results are listed in Table 2 and illustrated in Figure 1B. The largest areas of convergence for both meta-analyses were found in bilateral precuneus, with a slightly larger cluster in the left compared to the right precuneus. Further conjoined clusters of convergence were found in the left TPJ (angular gyrus and the posterior middle temporal gyrus) and the left middle occipital gyrus corresponding to the EBA.

### Region of interest based review

We followed-up the findings of our meta-analytic conjunction by a region of interest (ROI) based review. This approach does not include a statistical comparison. However, it gives an overview of which visual perspective taking studies contributed to the meta-analytic findings. We selected three peaks from our meta-analytic conjunction as ROIs: precuneus (*x* = 0, *y* = −53, *z* = 52), left dorsal TPJ/angular gyrus (*x* = −41, *y* = −59, *z* = 42) and left middle occipital gyrus (*x* = −49, *y* = −72, *z* = 13). ROIs were created by a 20 mm spherical volume around the peak coordinates. This radius corresponds to the size of the smoothing (FWHM) used by our meta-analysis. The other peaks from our conjunction analysis (left posterior middle temporal gyrus, right precuneus) did not enter our ROI analysis because they were located at too close distance to the three other ROIs, and were therefore practically not separable from them.

For each study, we checked if any of the reported activation coordinates fell within the 20 mm sphere around the three peak coordinates in the left precuneus, the left angular gyrus, and the left middle occipital gyrus. Table 3 summarizes the results of this review. It lists each study and indicates with a ‘+’ symbol if a study reported activation within a ROI. Contributions to the meta-analytic peak activation in the left angular gyrus were balanced over the three types of visual perspective taking: level 1 visual perspective (2/3 studies), level 2 visual perspective (3/5 studies), and level 2 viewer rotation (2/6 studies). Contributions to the peak activation in the left middle occipital gyrus were relatively weak for level 1 visual perspective (1/3 studies) and level 2 visual perspective (1/5 studies), but more substantial for level 2 viewer rotation (4/6 studies). Contributions to the meta-analytic peak in the left precuneus were relatively strong for level 1 visual perspective (3/3 studies), moderate for 2 viewer rotation (3/6 studies), and completely absent for level 2 visual perspective (0/5 studies).

## Discussion

We meta-analyzed brain activation for false-belief reasoning and visual perspective taking and looked for common brain areas engaged by these tasks with a conjunction analysis. We expected to find common activation in the left TPJ, based on our hypothesis that this area is implicated in processing perspective differences (Perner and Leekam, 2008). Our results confirm this expectation, as we found two clusters in the dorsal left TPJ (angular gyrus and posterior middle temporal gyrus) that were reliably engaged both in false belief and in visual perspective processing. In addition to these clusters, our meta-analysis revealed common areas in the left middle occipital gyrus and in the precuneus for false belief reasoning and visual perspective taking. In the next sections, we will discuss the potential functional roles of these locations of convergent brain activation.

### Left temporo-parietal junction

Our meta-analytic conjunction found two clusters of conjoint activation for visual perspective taking and false belief reasoning in the left dorsal TPJ, one in the angular gyrus at *z* = 42 and another one in the left posterior middle temporal gyrus at *z* = 32. No overlap in activation was found for right TPJ areas. These findings support the functional account of TPJ areas reviewed in our introduction (Perner et al., 2006; Perner and Leekam, 2008). In this view, the right TPJ is mostly responsible for belief-desire reasoning. Accordingly, our meta-analysis found activation only for false belief reasoning here, and no activation for visual perspective taking. The left TPJ, on the other hand, is thought to be involved in processing of alternative perspectives in a domain-general way. In support of this idea, we found an overlap in brain activation between visual perspective taking and false belief reasoning here.

An interesting aspect of the found overlap between visual perspective taking and false belief reasoning relates to its location within the left TPJ. Literature reviews have shown that different theory of mind tasks engage different parts of the left TPJ (Gobbini et al., 2007; Perner and Leekam, 2008; Bahnemann et al., 2010). For example, Perner and Leekam (2008) report that theory of mind tasks which require processing of a perspective difference—as for example the false belief tasks–engage more dorsal parts of the left TPJ, whereas theory of mind tasks that do not require such processing only engage more ventral parts located around the posterior Superior Temporal Sulcus (pSTS). This distinction is also relevant for the interpretation of David et al.'s (2008) study, which failed to find a functional overlap between visual perspective taking and theory of mind. David et al. (2008) used a preference judgment task to test theory of mind. Different from the false belief task, this task does not require processing of a perspective difference. Preferences are specific relations between a person and an object (e.g., “Max does not like apples”, “I do like apples,” but there is no difference in perspective, for Max and I have the same view. We both know that he hates apples and I like apples. Consequently, it becomes clear that—based on the functional distinction between dorsal and ventral pSTS made in literature reviews—one would not expect an overlap with visual perspective taking (in left dorsal TPJ). Whereas visual perspective taking should engage the left dorsal TPJ, the preference decision task should engage other areas more ventrally in the TPJ and in pSTS. Indeed, David et al. (2006) found activation for the preference decision task only in the right pSTS, and activation in more dorsal parietal areas for visual perspective taking. Conversely, the present meta-analysis looked at a theory of mind task that does present a perspective difference (false belief) and did find a functional overlap in the left dorsal TPJ with visual perspective taking.

**Figure 2:**
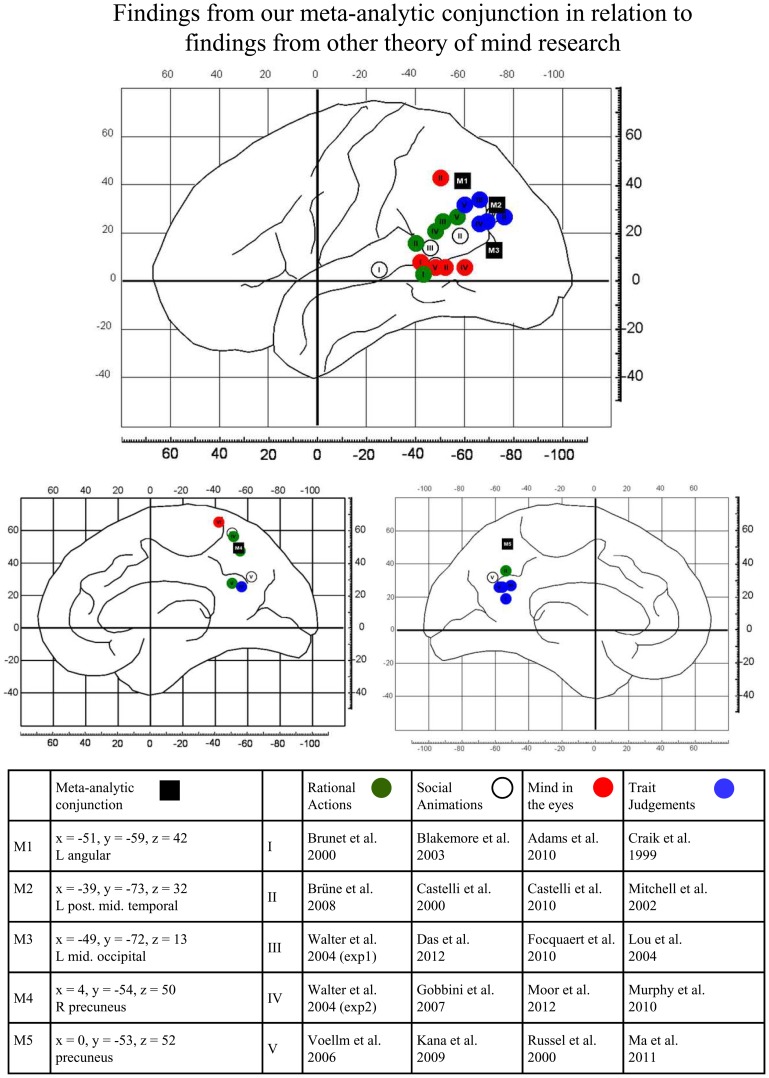
**Graphical illustration of the relations between the findings from our meta-analytic conjunction (black full rectangles) and findings reported in other theory of mind research**. Activation peaks reported in original studies are separately shown for tasks presenting rational actions (green full circles), social animations (white full circles), mind in the eyes (red full circles) and trait judgments (blue full circles). For details, see text.

To check whether the proposed functional distinction between dorsal and ventral TPJ can be linked to our observed activations for visual perspective taking, we performed an informal review which compares activation for different theory of mind studies to activation for visual perspective taking as found in our meta-analysis. In Figure 2, we indicate the results of our conjunction analysis between false belief reasoning and visual perspective taking by black boxes. In addition, we tentatively summarize temporo-parietal findings from popular theory of mind tasks by reviewing the peak-activations found in temporo-parietal areas for 5 studies per task-type. Green circles indicate locations for rational actions (Brunet et al., 2000; Walter et al., 2004; Voellm et al., 2006; Brüne et al., 2008). These tasks typically have a non-verbal format and present a cartoon-story about a person in the activation tasks. Participants are then asked about the goal of the person in the story, i.e., to predict what will happen next. In the control task, questions about non-mental aspects of the stories are asked (e.g., physical causality). White circles in Figure 2 indicate activation-peaks reported for social animations (Castelli et al., 2000; Blakemore et al., 2003; Gobbini et al., 2007; Kana et al., 2009; Das et al., 2012). These studies typically present video animations of simple geometrical shapes (see Heider and Simmel, 1944). In the activation condition, the animations portray actions which are typical for an intentional or social interaction. In the control condition, the animations show random or purely mechanical movements. For each movie, participants are asked to explain what is shown. Red circles in Figure 2 show activations for the so-called “mind in the eyes” tasks (after. The reviewed studies (Russell et al., 2000; Adams et al., 2010; Castelli et al., 2010; Focquaert et al., 2010; Moor et al., 2012) typically show in the activation task a photograph of a pair of eyes and ask which of two adjectives (e.g., *“concerned”* vs. *“unconcerned”*) best describes the mental state of the person. In the control tasks, again a photo of eyes is shown and participants are asked to indicate the gender of the depicted person.

Rational actions, social animations and mind in the eyes all do not require awareness of perspectives or processing of perspective differences for task performance. Consistent with Perner and Leekam's (2008) theorizing, Figure 2 shows that activations of these three task-types are mostly located ventrally and anteriorly to our conjunctions results. However, activation for another type of theory of mind task—judgments about another person's personality traits—shows some overlap with the dorsal TPJ areas identified by our conjunction analysis. Trait judgment tasks (Craik et al., 1999; Mitchell et al., 2002; Lou et al., 2004; Murphy et al., 2010; Ma et al., 2011) typically present personality trait-adjectives. In the activation task, participants are asked to indicate whether the adjective describes a particular person or not. In the control tasks, participants perform a non-mental state related task on similar trait words (e.g., *is this word written in upper- or lower-case?*).

As false-belief reasoning and visual-perspective taking, trait judgments may also require awareness of perspective, but for different reasons. Traits indicate habitual patterns of behavior, thought, and emotion. They are characteristic for a person when the person's habits deviate from the norm. For instance, a person is called “anxious” or “nervous” (Mitchell et al., 2002) if she tends to be concerned about situations where one normally has no reason to be anxious, i.e., the person takes a deviant perspective on how dangerous or challenging a situation is. Or a person is “stubborn” (Murphy et al., 2010) if she refuses to change her opinion or position on a subject when objectively (from the judging person's point of view) it is time to give up. So, many traits result from habitually biased perspectives, and trait judgments are judgments about whether a person habitually takes a different perspective on certain aspects of life.

### Precuneus

Although the precuneus is part of the typical set of brain areas active in theory of mind tasks (see e.g., Mar, 2011; Bzdok et al., 2012), relatively little has been said about its functional role in processing mental states of others. Several lines of research show that the area is implicated in mental imagery, i.e., the construction of a visual scene in absence of the appropriate external stimulus (Thomas, 2010). Studies found activation in the precuneus for the imagined execution of movements (e.g., Hanakawa et al., 2003), mental simulation of routes (Ghaem et al., 1997), mental imagery in deductive reasoning (e.g., Knauff et al., 2003) and for processing of intervals between tones in music perception (e.g., Platel et al., 1997). In their review on the precuneus, Cavanna and Trimble (2006) suggested that the main function of the precuneus in theory of mind is mental imagery to represent the perspective of another person. This function would be compatible with our finding that this area is engaged both in false belief reasoning and in visual perspective taking. Unexpectedly, however, we observed in our follow-up review that the precuneus tended to be engaged only by level 1 perspective and level 2 imagined viewer rotation tasks, but not by level 2 visual perspective taking tasks. Based on the assumption that activation in the precuneus reflects mental imagery to represent another's perspective, we would clearly expect activation also for level 2 visual perspective tasks. Contrary to that, we did not find such activation in any of the five reviewed level 2 visual perspective taking studies.

### Left middle occipital gyrus

Although activation in the lateral occipital cortex can be found for multiple forms of visual object recognition and visuo-spatial processing, we are particularly interested by the fact that our cluster in the left middle occipital gyrus is in good correspondence to the location of the EBA, with an euclidian distance of 5 mm to the coordinates reported in the seminal paper by Downing et al. (2001). The EBA was traditionally considered as a category-selective region for the visual processing of static images of the human body. Saxe et al. (2005) found that while the right EBA shows preferential activity for allocentric views on body-parts (i.e., the typical view we have on others), the left EBA is equally active for egocentric (i.e., the typical view we have on ourselves) and allocentric views on body-parts. Astafiev et al. (2004) found that the EBA is also engaged when participants perform movements (e.g., arm movement) in the absence of visual feedback. The authors interpreted these results as showing that in addition to a visual recognition function, the EBA is also engaged in maintaining our bodily representation by integrating visual, spatial attention, and sensory-motor signals. Recently, it has also been found that the EBA is engaged by imagined body movements. For example, Iseki et al. (2008) found activation in the EBA when participants were asked to imagine walking around in a room while they were actually lying in the fMRI scanner. (Deen and McCarthy, 2010) found activation in the EBA when participants read stories including passages about human movements (for example, ‘… on Christmas morning, Johnny ran down the stairs to the tree …’) compared to control stories (‘… Susan is sympathetic to children with disabilities …’).

Altogether, research on the EBA suggests that this area is involved in maintaining a bodily self-representation, and that this process is also engaged when one imagines a body movement of oneself or others. We speculate that activation in the EBA found in our meta-analysis may reflect imagined bodily transformations related to adopting a different visual perspective. We want to note that our follow-up review found that activation in the EBA mainly stemmed from level 2 imagined viewer rotation studies. This kind of task clearly invites imagining a movement of one's own body.

### Brain connectivity

To give a complementary characterization of our main findings, we take a look at their structural and functional connectivity profiles.

For a characterization of connectivity of the left dorsal TPJ, we refer to the work by Caspers et al. (2011) who present structural connectivity fingerprints from probabilistic fiber tract analyses for different parts of the left inferior parietal lobe. The activation peak from our conjunction analysis (*x* = −42, *y* = −59, *z* = 42) falls into the left angular gyrus and more precisely, in the cytoarchitectonic area PGa according to the Jülich Histological Atlas (Caspers et al., 2006, 2008) which is accessible with the software fslview (http://fsl.fmrib.ox.ac.uk/fsl/fslview/). The connectivity fingerprint for the area PGa is presented in Caspers et al. (2011, p. 371). In the left hemisphere, area PGa shows strong structural connectivity to (i) lateral prefrontal areas, in particular areas of the inferior frontal gyrus (ii) posterior occipito-temporal areas and posterior fusiform areas (iii) areas of the insula and (iv) parts of the superior parietal lobe. In addition, moderate connectivity is found to more anterior parts of the temporal gyrus and posterior cingulate gyrus/ventral precuneus.

For a characterization of precuneus connectivity, we rely on results from a recent resting-state functional connectivity analysis of this area (Zhang and Li, 2012). Results show that more dorsal parts of the precuneus are strongly linked to lateral occipital, superior parietal as well as lateral prefrontal areas in both hemispheres. More ventral parts of the precuneus are strongly linked to bilateral lingual gyri and the calcarine sulcus, bilateral inferior parietal lobuli (in particular the angular gyri) and the ventral mPFC. The activation peak from our conjunction analysis lies on the border between ventral and dorsal precuneus as defined by Zhang and Li (2012).

Taken together, connectivity data show that our three main findings, the left dorsal TPJ (corresponding to the angular gyrus and area PGa), the precuneus and the left middle occipital gyrus (roughly corresponding to the posterior occipito-temporal cortex) are structurally and functionally connected to each other. Via the precuneus, the left TPJ is also connected indirectly to the right TPJ, and from this perspective, it is evident that the left hemispheric network found in our meta-analysis is linked to a right hemispheric homologue network. Of particular interest, the connectivity fingerprint for the left TPJ area found in our conjunction analysis shows that this area is linked both to fronto-parietal areas (lateral prefrontal cortex, superior parietal lobe) which we only found in our meta-analysis of visual perspective taking, and to anterior temporal areas which we only found in our meta-analysis on false belief reasoning. It is tempting to speculate that this may reflect how a domain general function—processing of a perspective difference—can be applied to different problems (social versus spatial). However, direct evidence from task-based functional connectivity studies is needed to justify such a claim.

## Conclusion

To identify brain areas which are commonly engaged in social and visuo-spatial perspective taking, we performed a meta-analysis on false belief reasoning and visual perspective taking. False belief is a case of social cognition that requires processing of a perspective difference to understand someone's mistaken belief about the world which contrasts with reality. We found common activation for false belief reasoning and visual perspective taking in the left but not right dorsal TPJ. This fits with the idea that the left dorsal TPJ is responsible for representing different perspectives in a domain-general fashion (e.g., Perner and Leekam, 2008). In addition, we found common activation for false belief reasoning in the precuneus and the left middle occipital gyrus. Common activation in the precuneus can be linked to mental imagery which may support both social and visuo-spatial scene construction, whereas common activation in the left middle occipital gyrus—falling into the EBA–can be linked to imagining a change in one's body position in order to get another's point of view.

## Author contributions

Matthias Schurz, Josef Perner and Markus Aichhorn designed and planned this work. Matthias Schurz and Anna Martin implemented the meta-analysis. Matthias Schurz and Josef Perner wrote the manuscript.

### Conflict of interest statement

The authors declare that the research was conducted in the absence of any commercial or financial relationships that could be construed as a potential conflict of interest.

## Footnotes

^1^Viewer rotation' refers to an imagined change of one's own point of view, i.e., imagining oneself rotating around an object in space, arriving at a new viewpoint on it.

^2^A recent level 1 visual perspective taking study (Ramsey et al., 2013) did not look at the contrast other > self, but at interactions between perspective taking (self vs. other) and the consistency of perspectives (i.e., do self and other perspectives differ in the task-response that they require?). Ramsey et al.'s (2013) results show that the consistency between perspectives is an important and previously ignored determinant of brain activation. However, these data go beyond the scope of the present meta-analysis and cannot be synthesized with results from other studies in our meta-analysis. Therefore, we did not include Ramsey et al.'s (2013) study in our analysis.
